# Electric Scooter-Related Injuries: A New Epidemic in Orthopedics

**DOI:** 10.3390/jcm10153283

**Published:** 2021-07-25

**Authors:** Alexandre Coelho, Pablo Feito, Laura Corominas, Juan Francisco Sánchez-Soler, Daniel Pérez-Prieto, Santos Martínez-Diaz, Albert Alier, Joan Carles Monllau

**Affiliations:** 1 Orthopaedic Department, Parc de Salut Mar, Universitat Autònoma Barcelona, 08003 Barcelona, Spain; pfeito@psmar.cat (P.F.); jfsanchez@psmar.cat (J.F.S.-S.); Dperezprieto@hospitaldelmar.cat (D.P.-P.); smartinezd@psmar.cat (S.M.-D.); aalier@psmar.cat (A.A.); jmonllau@psmar.cat (J.C.M.); 2 Department of Orthopaedics and Traumatology, Hospital Sant Joan de Déu, Passeig de Sant Joan de Déu, 2, Esplugues de Llobregat, 08950 Barcelona, Spain; lcorominas@hsjdbcn.es; 3 IMIM (Hospital del Mar Medical Research Institute), 08003 Barcelona, Spain; 4 Orthopaedic Department, ICATME-Institut Universitari Quirón-Dexeus, Universitat Autònoma Barcelona, Sabino de Arana, 5-19, 08017 Barcelona, Spain

**Keywords:** electric scooters, accidents, epidemiology, incidence, injury, prevention

## Abstract

Background: The use of electric scooters has increased considerably as they are an accessible means of transportation. The number of injuries from falls and collisions has risen significantly. Therefore, the aim of the study was to describe demographics injury patterns of electric scooter accidents produced over one year. Methods: A prospective observational study of electric scooter- related injuries presented in the emergency room from May 2019 until May 2020. The inclusion criteria was based on the direct cause of injury produced while an electric scooter was in use. Demographic data, the use of a helmet or the lack thereof, accident mechanism, injury time, type of injury produced, and the treatment applied were collected. Results: Over the study period, 397 patients were identified with a total of 422 injuries. The mean age was 30.8 years, with 12.6% of patients being minors. The patients mainly presented in evening hours and in summertime at the emergency department. Of the total injuries seen, 46.9% were fractures. Some 25% of the total cases required surgery. Only 19% of the riders wore a helmet at the time of the accident. Most of the fractures were to the upper limbs (62.6%). There was a greater incidence of radius fractures. Conclusion: Injuries incurred while using electric scooters are an emerging phenomenon, despite existing regulations. In this study, most injuries occurred in young men and were due to falls from the vehicle. Nearly half of those injuries were fractures to the upper limbs. Surprisingly, 50% of the fractures required surgery.

## 1. Introduction

The way we move around the city is currently undergoing change. We are increasingly opting for more practical personal vehicles, which are easily accessible and give us the autonomy to move where and when we want. The electric scooter (e-scooter) is trending and is one of the most widely used means of transportation in the world. In Spain, it all started with a renting scheme in September 2017 in Madrid and Barcelona that rapidly spread around the country [1].

These devices can reach a speed of 30 km/h and even greater when moving downhill. It is powered by a rechargeable battery that can last 6–8 h depending on the model [2]. Since it is cheaper than other means of transportation such as cars or motorbikes and it can travel in bike lanes to avoid the usual city traffic, it has become one of the most preferred means of urban transport due to its accessibility and facility of use [3].

However, this scootermania has a cost. The number of injuries associated with e-scooter accidents has become a public health issue by causing a massive increase of admissions in emergency rooms [4,5,6]. This study aims to describe the demographics and injury patterns in patients attended to in the orthopedics emergency room after an e-scooter accident over 1 year in a hospital in Barcelona, Spain.

## 2. Materials and Methods

A prospective observational study was designed including all patients admitted to the Hospital del Mar emergency room due to an injury produced after e-scooter accidents between May 2019 and May 2020. The hospital is a 3rd level center located within the southern area of the city of Barcelona that treats approximately 18,000 patients per year in the emergency department.

Injuries produced after falls from electric skateboards, electric bicycles and self-balancing scooters (e.g., Segway^®^, Bedford, NH, USA.) were excluded. Cranial and organ injuries were not included in this study as they are admitted to other emergency departments like neurosurgery and general surgery.

Demographic data such as age, gender and nationality were collected. Patients were also asked about the mechanism (fall, collision, hit by vehicle) and time of injury as well as whether they wore a helmet or not at the time. Incidentally, they were asked if they were driver’s license holders. The investigators also collected data about the type of injury, the affected side of the body, its location, the radiological tests performed, and treatment applied. Institutional Review Board Approval was obtained before beginning the study (CEIm 2020-9669).

### Statistical Analysis

The statistical analysis was performed using SPSS version 16 (SPSS Inc., Chicago, IL, USA) software package. Categorical data was presented as frequencies and percentages, while continuous data was presented as mean ± standard deviations (SD). The *p*-values for categorical data were obtained from the chi-square or Fisher’s exact test.

## 3. Results

A total of 397 patients presented in the emergency room with injuries related to e-scooter accidents during the study period. There were 238 male (59.9%) and 159 females (40.1%) with a mean age of 30.8 years (SD, 10.1). The demographics and accident characteristics collected are shown in Table 1.

**Figure 1:**
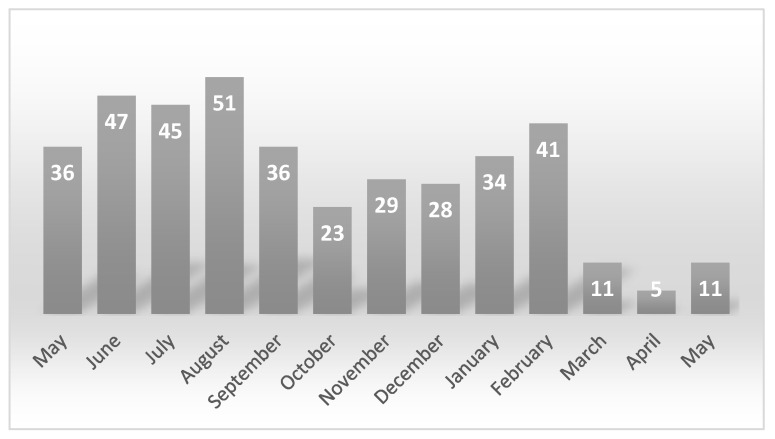
Most injuries were produced during summer months. A decrease in the number of injuries can be observed during the COVID-19 lockdown period.

The patient breakdown was 374 (94.2%) e-scooter riders, 18 pedestrians, 1 rider of another type of vehicle and 4 e-scooter passengers. Only 55 patients (19.1%) were wearing a helmet at the time of injury. In most of the cases, the mechanism of injury was a fall from the vehicle (341 (85.9%)). That was followed by 38 (9.6%) collisions between vehicles and 18 (4.5%) between an e-scooter and a person. Of all the injured, 300 (75.6%) were residents and 97 (24.4%) were tourists. Most of the injuries (242 (61%)) occurred in the afternoon period (3 pm−11 pm). The vast majority of the injuries occurred in summer (179), from June to September, with a notable decrease from March 2020 to May 2020, as observed in Figure 1.

A total of 422 injuries were evaluated. Considering that a patient could present different patterns of injury at the same time, that total covers 171 contusions (40.5%), 32 (7.6%) wounds requiring stitching, 21 (5%) dislocations and 198 (46.9%) fractures. There were 191 (45.2%) injuries on the left side of the body, 202 (7.9%) on the right side and the remaining 29 (6.9%) injuries were to the axial skeleton. Most of the injuries (235, 55.7%) were to the upper limbs and most of the accidents occurred during evening hours with a total of 242 (61%) patients admitted to ED between 3 pm and 11 pm. Relative to the fractures, most were to the upper limbs (124, 62.6%), being radial fractures the most common (77, 38.9%).

**Figure 2:**
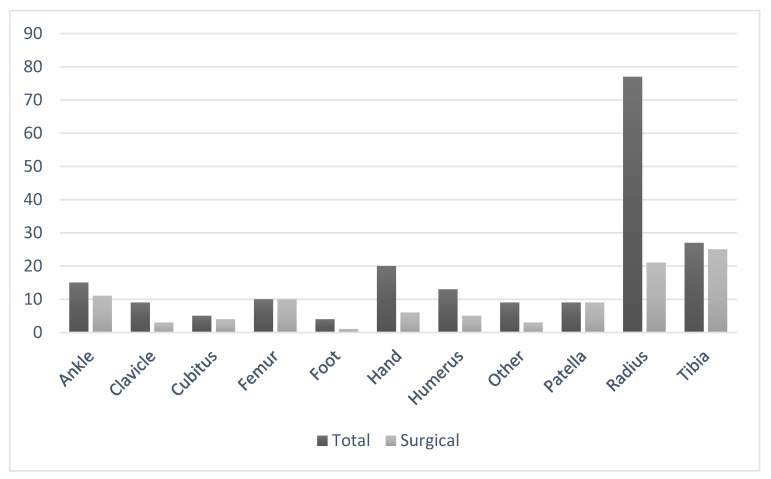
Although most fractures were produced on radius, lower limb fractures were more commonly treated by surgery. In ‘other’ fractures were included 1 acetabular injury, 4 costal fractures, 2 vertebral fractures and 2 mandibular fractures.

As for the treatment of fractures, 98 (49.5%) needed surgical treatment and 100 (50.5%) were treated conservatively, as can be seen in Figure 2. Lower limb fractures (66) were more commonly treated with surgery (57, 86.4%) than upper limb fractures. Of the 124 upper limb fractures, 39 were treated surgically. Two mandible fractures were treated surgically, and the rest of axial fractures were treated conservatively.

## 4. Discussion

E-scooters are a practical and convenient means of transport. However, their use is accompanied by an increasing number of emergency room visits due to accidents over recent years. It is becoming a major public health issue, due to the associated morbidity and costs related to injuries produced after accidents. In this study, it was observed that 46.9% of the injuries related to e-scooter accidents resulted in fractures and 49.5% of them required surgery for their treatment, which sustains that these types of accidents may be managed as high-energy injuries. According to our results, only 18.9% of patients wore a helmet in the moment of the accident, which seems like riders are not completely aware of the potential risks of driving an e-scooter [5,7,8].

Due to the high prevalence of accidents and risky behaviors associated with e-scooters, appropriate regulatory responses have been published that include an increase in the number of fines applied [9]. In the latest regulation published in Spain (2017), it has been established that only riders over 16 years old with only one rider per vehicle are authorized in a public space. Furthermore, the use of lights, a helmet and a bell are required. In addition, the maximum velocity allowed is limited to 30 km/h. Any infringement is punishable with a fine up to €500, depending on the severity of the infraction.

In our study, 24.7% of the patients referred to the emergency department required surgical treatment. That fact along with the scarce data published on e-scooter injuries supports them being treated as high-energy mechanisms that can result in severe injuries. These results are in line with those published by Ishmael et al. and Störmann et al. in which 27.6% of injuries were treated surgically [3,10]. It is important that riders understand the hazards associated with moving around on a two-wheeled vehicle that may reach 30 km/h. E-scooters not only share lanes with other vehicles such as bicycles, motorcycles or cars but also sidewalks with pedestrians due to the lack of laws on circulation. Additionally, wheel thickness makes e-scooters vulnerable to different obstacles in roads that may lead to falls. Although most of the fractures are to the upper limbs, surgical procedures are more frequently required on the lower limbs. This may be causally related to the speed at the time of the accident as higher speeds diminish the reaction time. The fall-damping reaction with the upper limbs may also explain why most injuries are to the upper limbs when compared to skateboard-related injuries [11]. In fact, the incidence of fractures produced after e-scooter accidents is comparable to the reported in skateboard accidents—Lustenberger et al. reported an incidence of 50.3%—and superior to the reported in bicycle accidents—Davidson et al. and Rivara et al. reported an incidence of 40% and 32.6%, respectively [12,13,14]. The incidence of fractures observed in e-scooter related accidents was superior to the incidents observed in road bicycle (23%) and mountain bike injuries in USA (27–43%) [15,16]. When comparing fracture incidence in traffic accidents in Sweden, Meredith et al. reported that car accidents resulted in fracture in 37% of cases, motorcyclists in 27.6% and bicyclists in 15.6% [17]. Even though mechanisms of injury differ between vehicles, this high percentage of fractures observed in e-scooter accidents when comparing to other means of transportation reinforce the need to develop safety measures to avoid severe lesions.

Although legal mechanisms are in force, most riders underestimate the dangers involved with e-scooter use as only 16.8% of patients wore a helmet at the moment of the accident. That percentage of riders using a helmet is similar to the trend reported by Trivedi et al. of 4.4% and Liew et al. of 5.6% [5,18]. Although it is well known that helmet use significantly reduces the risk of brain injury and long-term complications in motorcyclists, there is limited data about its use with e-scooters [19]. La Torre et al. conducted a study comparing head injuries before and after implementing a helmet law for scooter riders and found a relative risk reduction of 0.42–0.44 [20]. In 2018, Høye also reported that the use of bicycle helmets reduced head injuries by 48%, meaning an overall 60% reduction in severe head injuries and 28% reduction in facial injuries. This reduction may be even greater in situations of increased crash risk such as on slippery or ice roads [21].

Most emergency department presentations were observed in the evening hours (61%). These results are in line with those published in recent literature in which 60 to 65% of accidents were produced during the late evening hours [10,22]. Trivedi et al. reported injuries produced from 3 pm to 11 pm in some 56% of the cases [5]. It may be because e-scooters are more frequently used as a recreational means of transport rather than a means of transportation to the workplace. The finding herein is that there was no difference in the fracture incidence between the morning (50.5% of total injuries produced), evening (49.2%) and nighttime (45.6%) hours. This result is similar to those reported by Moftakhar et al. [23]. Additionally, a greater number of accidents were seen during the summer. It is probably due to an increase in the recreational use of e-scooters. A notable decrease was also noticed from March 20th to May 20th, 2020. It is the period in which a quarantine period was declared due to the global pandemic of SARS-COV-19, suggesting its effect on the incidence of e-scooter accidents per year.

According to our findings, 14.7% (58) of patients attended to in the emergency room were underage. Some 76% of those were under 16, which is the minimum legal age allowed to ride an e-scooter [24]. Of those 58 patients, only 3 (5.1%) wore a helmet, showing that children and parents are unaware of the risks associated with the e-scooter. Previous studies concluded that the use of a helmet reduces the risk of a head injury and that the most effective way to reduce accident-related injuries is to use protective gear [11,25]. It was also observed that 38% of the injuries produced in children required orthopedic treatment, either with a cast (27.6%) or surgery (10.4%). Children are not only at risk as users but also when they are carried by their parents without any protective equipment or security accessories.

While e-scooters are becoming more popular and their use is growing, it is important to highlight the risks and reinforce suggestions for their proper use. Helmet use should be mandatory to prevent head and neurological injuries, with an expected decrease of brain injuries of some 60% [21,26]. Moreover, the use of knee and elbows pads should be promoted to prevent severe injuries to the extremities as per scooter, skating and extreme sports safety recommendations [27,28]. Another strategy to improve e-scooter safety may be to improve rider education such as the need for a license to circulate with these vehicles [29]. Kosola et al. reported that a mandatory helmet law and a driver’s license requirement reduced the number and severity of moped- and scooter-related injuries [30]. Finally, we would also reinforce that sidewalk circulation should be forbidden since almost 5% of patients arriving in the emergency department were hit by e-scooters.

To the best of our knowledge, this is the first prospective study of e-scooter-related injuries in Europe and the first study related to the e-scooter published in Spain. Since Trivedi et al. published the first descriptive study about e-scooter-related injuries, interest around accidents produced by this means of transportation has increased [5]. Table 2 provides a comparison of the results of this study with previous literature. One of the main strength points of this study is the sample size obtained, one of the biggest sample sizes reported in literature, which may provide an accurate description of non-head injuries after e-scooter accidents. In addition, the study was designed for a duration of one year, which allows observing differences in the number and pattern of injuries in different periods of the year. When analyzing the results of this study and the data previously reported, it can be observed that a high percentage of the injuries produced are fractures and, therefore, allow us to understand these types of casualties as high-energy accidents. It is also alarming that the vast majority of riders do not wear a helmet when driving e-scooters, which can lead to severe head injuries, preventable with the use of this protective gear. Finally, it is also noteworthy that up to 15% of electric scooter accidents occur in minors, an age in which driving of motorized vehicles is not allowed. With this article, the authors intend to raise awareness of the risks of using e-scooters, recognizing that this type of vehicle is a valid alternative for intra-urban mobility as long as the precautionary measures are taken: helmet and protective equipment use, avoid driving on sidewalk and respect traffic rules. 

Our study has several limitations. The data for head and torso injuries produced after e-scooter accidents was not collected as it is evaluated by neurosurgery, thoracic surgery or general surgery based on the Severity Index. This limits the possibility to relate a head injury with helmet use and to describe the prevalence of head injuries after e-scooter accidents. Moreover, only patients attended in our hospital were included, which may limit the evaluation of geographic and urban planning that may influence the frequency, severity and characteristics of injuries produced in Barcelona. Another limitation is that the study was performed only over one year, and it was a year in which there was an unexpected quarantine due to the epidemic of SARS-COV-19 that may have brought down the number of accidents and injuries produced. Although the study and patient inclusion process were finished in May 2020, an upturn in the number of e-scooter-related accidents has been observed from June 2020 onward.

## 5. Conclusions

Injuries caused by electric scooters are an emerging phenomenon despite existing regulations. Most injuries are to the upper limb and a significant part of them are fractures. A striking 25% of the injuries seen required surgery. E-scooter-related injuries may be severe and preventable. Current regulations might not be enough to aid in preventing these accidents. Enhanced user education, stricter laws and the need for protective gear are called to bring down the incidence of serious accidents with e-scooters.

## Figures and Tables

**Table 1:** Patient and accident characteristics associated with e-scooter-related injuries during a 1-year study period.

	N (%)
**Age**	
<16	50 (12.6%)
16–25	102 (25.7%)
26–40	166 (41.8%)
41–64	74 (18.6%)
>64	5 (1.3%)
**Gender**	
Male	238 (59.9%)
Female	159 (40.1%)
**Nationality**	
Resident	300 (75.6%)
Tourist	97 (24.4%)
**Injured patient**	
Rider	374 (94.2%)
Non-rider	23 (5.8%)
Pedestrian	18 (4.5%)
Carried by rider	4 (1%)
Other vehicle	1 (0.3%)
**Helmet use**	
Yes	55 (19.1%)
No	234 (80.9%)
**Driver’s license**	
Yes	162 (54.9%)
No	133 (45.1%)
**Mechanism of injury**	
Fall	341 (85.9%)
Collision	38 (9.6%)
Hit by a vehicle	18 (4.5%)
**Time of injury**	
7 am–3 pm	87 (21.9%)
3 pm–11 pm	242 (61%)
11 pm–7 am	68 (17.1%)

**Table 2:** Comparison of previous studies reporting injuries after e-scooter accidents.

Author	Study Design	Country	Sample Size	Mean Age	Riders	Helmet	Minors	Fractures	Most Frequent
Trivedi 2018 [5]	Retrospective	USA	249	33.7	91.6%	4.4%	10.8%	31.7%	Upper extremity
Blomberg 2019 [31]	Retrospective	Denmark	130	27	86.6%	3.6%	10%	11.5%	Face
Campbell 2019 [4]	Retrospective	New Zealand	21	31	N.S.	N.S.	14.3%	100%	Ankle
Dhillon 2020 [32]	Retrospective	USA	87	35.1	N.S.	18.4%	N.S.	57.4%	Face
Ishmael 2020 [3]	Retrospective	USA	73	35.4	97.3%	N.S.	5.5%	93.2%	Lower extremity
Liew 2020 [18]	Retrospective	Singapore	36	34	38.9%	5.6%	N.S.	33.3%	N.S.
Moftakhar 2020 [23]	Retrospective	Austria	175	34.4	94.9%	N.S.	N.S.	35.2%	Upper extremity
Puzio 2020 [22]	Retrospective	USA	92	30	N.S.	0%	N.S.	24%	N.S.
Stormann 2020 [10]	Retrospective	Germany	76	34.3	92.1%	1.3%	2.6%	48.6%	Upper extremity
Bloom 2021 [33]	Retrospective	USA	248	35.8	N.S.	3%	9%	21.4%	Radius
Coelho 2021	Prospective	Spain	397	30.8	94.2%	19.1%	12.6%	46.9%	Radius

N.S.: Non specified.
